# Versatile Hydrogel Based on a Controlled Microphase-Separation Strategy for Both Liquid- and Solid-Phase 3D Printing

**DOI:** 10.1021/acsnano.4c08896

**Published:** 2024-10-30

**Authors:** Qirui Wu, Yidan Xu, Songjiu Han, Anbang Chen, Jiayu Zhang, Yujia Chen, Xiaoxiang Yang, Lunhui Guan

**Affiliations:** † State Key Laboratory of Structural Chemistry, Fujian Key Laboratory of Nanomaterials, and CAS Key Laboratory of Design and Assembly of Functional Nanostructures, Fujian Institute of Research on the Structure of Matter, Chinese Academy of Sciences, Fuzhou, Fujian 350108, China; ‡ School of Mechanical Engineering and Automation, Fuzhou University, Fuzhou 350108, China; § Department of Oncology, The First Affiliated Hospital of Anhui Medical University, Hefei 230000, China

**Keywords:** microphase-separation, 3D printing, flexible devices, low-hysteresis, hydrogels

## Abstract

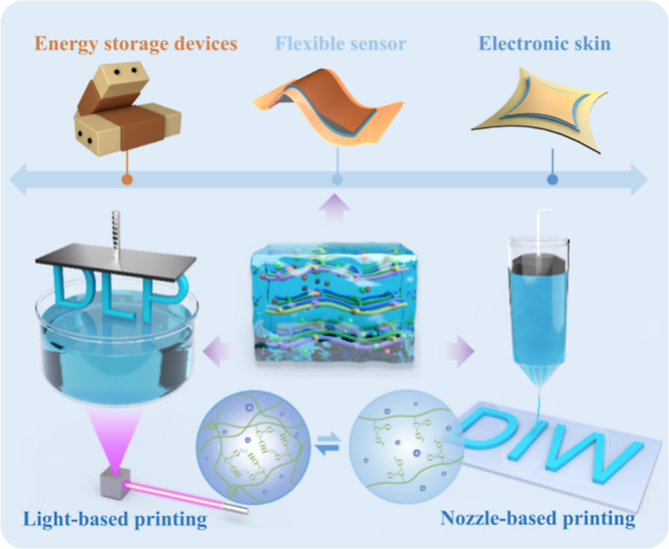

Hydrogels are considered indispensable materials for fabricating flexible devices with their excellent flexibility and workability. To efficiently transform hydrogels into flexible devices, three-dimensional printing technology offers a powerful approach. However, hydrogels suitable for a single printing strategy have proven inadequate for fabricating flexible integrated devices. Herein, we report a simple and two-phase 3D-printed hydrogel (TP-3DPgel) achieved through a controlled microphase-separation strategy. The microphase-separation regions can undergo reversible changes through pH adjustment, giving TP-3DPgel an extremely broad viscosity tuning range from liquid to solid states. This overcomes limitations imposed by extreme rheological properties in different 3D printing processes, making this ink suitable for both liquid-phase digital light processing (DLP) 3D printing and solid-phase direct ink writing (DIW) 3D printing. Simultaneously, the TP-3DPgel exhibits excellent mechanical properties, including high stretchability (>1100%), high strength (0.82 MPa), low hysteresis (∼5.4%), and fatigue resistance. Moreover, TP-3DPgel exhibits high-resolution 3D printing capabilities, making it suitable for both DLP and DIW-3D printing to achieve high-quality fabrication from 2D filaments to 3D structures. Interestingly, we utilized both DIW and DLP-3D printing to fabricate various functional flexible devices, including energy storage devices, sensors, and electronic skins, showing in detail the outstanding compatibility and processability of TP-3DPgel, which offered a reliable strategy for 3D printing functional devices.

Flexible devices, such as wearable electronics, energy storage devices, electronic skin, and flexible robots, had gained significant attention as indispensable media for human–machine interaction and the internet of things in the artificial intelligence era.^1−6^ Among them, hydrogels were ideal candidates for the fabrication of flexible devices due to their inherent flexibility and workability. Obviously, the fabrication of advanced flexible devices required compatible flexible materials and processing technologies.^7−10^ Due to the limitless size and flexibility of 3D printing (also known as additive manufacturing), which enabled complex topological designs of materials, it is widely used in the manufacturing of soft intelligent devices.^11−15^ Currently, the structured design of hydrogel materials through 3D printing to create customized functional devices has been widely reported.^16−20^ For example, synaptic structures mimicking Meissner corpuscles, suction cup structures inspired by octopus tentacles, and fiber structures inspired by spider legs have all been developed using digital light processing (DLP) or direct ink writing (DIW) printing strategies.^21−28^ However, hydrogels compatible with a single printing strategy prove to be insufficient for fabricating integrated devices with the increasing demand for multimodal flexible devices. This limitation has become a significant challenge, hindering the practical application of flexible integrated devices.

Specifically, DLP-3D printing achieved the fabrication of 3D structures through selective photopolymerization at the interface between the platform and the ink, which was a typical top-down fabrication strategy. Throughout this process, the ink needed to maintain good flowability and a certain level of transparency. However, the manufacturing of flexible devices often requires the addition of additional materials (such as carbon-based materials, conductive polymers, metal oxides, etc.) to impart various functionalities.^29^ This typically disrupts the ink’s initially stable rheological properties, leading to printing failures due to excessively high ink viscosity. Similarly, most of these introduced additives were dark colors (e.g., black for carbon materials and conductive polymers), absorbing specific wavelengths of light, which causes the ink to lack sufficient light energy for efficient curing.^30^ Therefore, the stringent requirements of ink in DLP-3D printing have become a significant barrier to its application in the fabrication of flexible devices. Interestingly, despite its demanding ink requirements, DLP-3D printing has remained highly sought after by many scholars due to its fast-printing speed, high resolution, and high degree of freedom. DIW-3D printing was based on a micronozzle inkjet mechanism that extruded semisolid ink along predefined paths for shaping.^31−34^ This bottom-up manufacturing method does not require consideration of the internal phase composition of the ink system, making it suitable for a wide range of composite ink formulations.^35^ Nevertheless, the shape retention capability of the ink extruded from the nozzle tip had always been challenging, especially when materials were distributed around the periphery of the built layers, which can significantly affect the resolution of the formed structure. Therefore, inks suitable for DLP and DIW-3D printing exhibited markedly different intrinsic properties. The former required inks with very low viscosity, while the latter prefers semisolid gel materials with a certain level of viscosity. In short, DLP and DIW-3D printing each play to their strengths in constructing microscale structures for flexible devices. For example, DLP-3D printing was preferred for fabricating conical, cylindrical, and pyramidal synaptic structures on scales ranging from 50 to 100 μm. However, inks doped with materials such as carbon nanotubes or poly(3,4ethylenedioxy thiophene):polystyrenesulfonate (PEDOT:PSS) had higher viscosity and lacked the transparency necessary for photopolymerization, making DIW 3D printing more suitable. Currently, there are no reported inks suitable for both printing methods. This difference in ink requirements acts like a “language barrier”, limiting the interaction and development between the two 3D printing technologies. Especially with the rising demand for multimodal flexible integrated devices, a single printing method cannot fully meet manufacturing requirements. Therefore, developing an ink compatible with both 3D printing methods was imperative.

Herein, we report a versatile two-phase 3D printing hydrogel (TP-3DPgel) ink based on a controlled microphase-separation strategy, which was suitable for both liquid-phase DLP-3D printing and solid-phase DIW-3D printing. The viscosity range of the hydrogel ink spans over 6 orders of magnitude (10^–2^ to 10^4^ Pa s^–1^). The entire process could produce hydrogel ink precursors with different degrees of microphase-separation simply by adjusting the pH. Simultaneously, TP-3DPgel exhibited excellent low hysteresis (>5.4%) and fatigue resistance, which endured up to 100 cycles of repeated loading and unloading under large strain with negligible hysteresis effects. Additionally, TP-3DPgel exhibited high-precision multiscale 3D printing characteristics, with a resolution spanning from 50 to 510 μm, which enabled the fabrication of both 2D filaments and 3D objects through DLP and DIW-3D printing, respectively. Surprisingly, TP-3DPgel demonstrated outstanding compatibility and processability, allowing it to be mixed with conductive polymers, nanoparticles, and carbon materials as inks without compromising the precision of DIW-3D printing. Taking PEDOT:PSS as an example, it has enabled the design and DIW-3D printing of structures ranging from supercapacitors to omnidirectional strain (OS) sensors and temperature sensors. Furthermore, we capitalized on the high-resolution 3D printing capabilities of TP-3DPgel to demonstrate the integrated manufacturing of high-sensitivity e-skin arrays by DLP printing microridged piezoelectric sensors. In summary, our innovative design of TP-3DPgel as a versatile 3D printing ink provided potential momentum for the development of additive manufacturing and flexible devices.

## Results and Discussion

**Figure 1:**
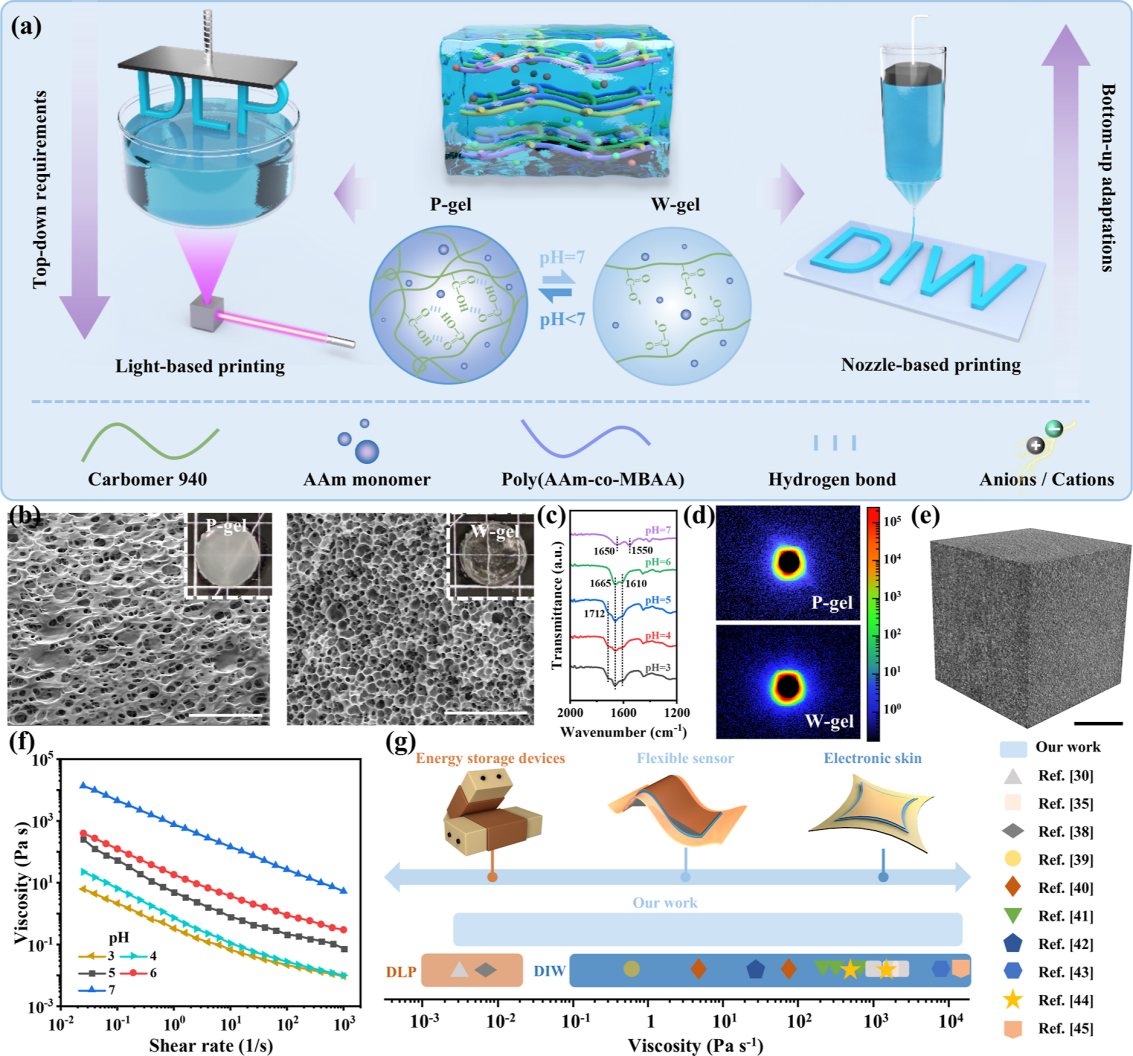
Design concept and intrinsic characteristics of controllable microphase-separated TP-3DPgel. (a) Material composition of TP-3DPgel and the schematic diagram illustrating its application in DLP and DIW-3D printing. (b) P-gel and W-gel SEM images and corresponding optical images. Scale: 10 μm. (c) FT-IR spectra of TP-3DPgel at different pH values. (d) SAXS patterns of P-gel and W-gel. (e) 3D Nano CT images of P-gel based on differences in density. Scale: 100 μm. (f) Relationship between the viscosity of TP-3DPgel at different pH values and shear rate. (g) Comparison of viscosity between TP-3DPgel and different 3D printing inks.

As shown in Figure 1a, we designed a two-phase 3D printable hydrogel (TP-3DPgel) ink based on a controlled microphase-separation strategy. This ink is capable of simultaneously satisfying both nozzle-based and light-based printing, aiding in overcoming the “language” barriers of different additive manufacturing technologies. Specifically, precursor inks require a rational design to meet the requirements of different 3D printing architectures. For example, the DIW-3D printing method, which involves extruding gel through a nozzle, requires the precursor ink to exhibit typical viscoelastic behavior, including high viscosity, shear yield stress, thixotropy, and significant shear-thinning behavior. The ensures its broad ink adaptability during the bottom-up printing process. On the contrary, digital light process (DLP)-3D printing based on ultraviolet curing strategy imposes requirements such as high fluidity, transparency, and curing efficiency on precursor ink. This created conflicting demands compared to those of DIW-3D printing, thereby restricting the development of additive manufacturing for flexible electronic devices. Fortunately, the synthesized TP-3DPgel achieved controlled microphase-separation through simple pH adjustment, resulting in different rheological behaviors that were perfectly adapted to various 3D printing techniques. This was primarily due to the unique phase composition of the TP-3DPgel. First, we selected neutral acrylamide as the monomer and *N*,*N*′-methylene bis(acrylamide) (MBAA) as the cross-linker, dissolving them in a carbomer solution. Additionally, the introduction of a moderate amount of calcium chloride imparts electrical conductivity to TP-3DPgel (Figure S1). Through in situ polymerization, a highly entangled cross-linked interpenetrating network (IPN) system was formed. Among them, carbomer was a high-molecular-weight polymer with carboxylic acid groups. In the initial state, the carboxyl groups on the polymer chains form coiled clusters through intramolecular hydrogen bonding, creating microphase-separated regions within the hydrogel. Subsequently, deprotonation of the carbomer polymer chains occurred by adjusting the pH with a basic compound to achieve neutrality. This deprotonation process promoted the dissociation of the clustered molecular chains due to the repulsive forces of the carboxylate anions, resulting in a highly transparent and viscous ink (Figure S2). Therefore, the highly entangled network structure and the interactions among the functional groups within the molecular chains promoted the construction of microphase-separated regions. This was reflected in the macroscopic change of TP-3DPgel from opaque to transparent, accompanied by a transition from a liquid phase to a solid phase. Interestingly, this process can be controlled by adjusting the precursor’s pH, allowing the macroscopic morphology of TP-3DPgel to undergo reversible changes (Figure S3). Figure S4 shows the optical images of TP-3DPgel at different pH values. Notably, the introduction of hydrochloric acid exacerbated the clustering of polymer chains in the hydrogel, further worsening the opacity. Therefore, TP-3DPgel exhibited opacity under acidic conditions, which was attributed to interfacial reflections and the difference in refractive indices between the two phases caused by the presence of microphase-separated regions. In contrast, neutral TP-3DPgel, with its uniform microstructure, showed greater light penetration and was completely transparent. For the sake of clarity in subsequent discussions, we referred to the initial state (pH = 5) of TP-3DPgel as P-gel and the neutralized state(pH = 7) as W-gel.

To verify the aforementioned microphase-separation phenomena and mechanisms, the microstructural differences between the P-gel and W-gel were analyzed using scanning electron microscopy (SEM). As evident in Figures 1b and S5, P-gel exhibited a porous scaffold with clearly defined microphase-separated regions, while W-gel showed a uniform and loosely porous network structure, which confirmed that pH adjustment causes changes in the internal tissue structure of the hydrogel. Further insights into the transformation of different component phases were revealed through FT-IR spectroscopy. As featured in Figures 1c and S6, the conversion of TP-3DPgel from P-gel to W-gel was accompanied by the disappearance (1712 cm^–1^) and shift (1665 and 1610 cm^–1^) of characteristic peaks, which further confirmed the dissociation of clustered –COOH groups on the polymer chains through hydrogen bonding interactions and the strong repulsion between anionic –COO^–^ groups.^36,37^ Additionally, the charge distribution of the hydrogels was assessed by measuring the zeta potentials of both P-gel and W-gel (Figure S7). The results indicated that the transition from acidic P-gel to neutral W-gel significantly increased the electronegativity of W-gel due to the presence of abundant carboxylate anions. This finding further corroborates our hypothesis regarding the formation of microphase separation. SAXS results indicate distinct diffraction patterns for the P-gel and W-gel (Figure 1d). W-gel exhibits a circular scattering pattern, whereas P-gel shows a pronounced elliptical scattering pattern. Furthermore, the scattering intensity distribution reveals that the signal for W-gel is more compact with a gradual decay in intensity, while the signal for P-gel exhibits more pronounced decay at the periphery (Figure S8). This suggests that W-gel has a denser or more uniform structure, while P-gel has larger disordered regions or a looser structure, which is consistent with the SEM results. In order to accurately investigate this phenomenon at the microscale, we employed in situ nondestructive nanocomputed tomography (Nano CT) to visualize and construct models of the internal microstructure of the phase-separated hydrogels. Figure 1e presents the 3D reconstruction of P-gel, revealing a distinct solid–liquid phase interface between the solvent phase (colored dark) and the polymer phase (colored gray) within the hydrogel. This demonstrated the presence of a denser solid polymer-rich region, providing strong evidence for the existence of phase separation and supporting the aforementioned hypothesis.

We obtained precursor materials with different rheological properties through microphase-separation strategies to adapt to solid–liquid phase 3D printing. Therefore, steady-state flow experiments were conducted to elucidate the viscosity of the ink at different pH values, allowing for a quantitative analysis of the rheological properties of the TP-3DPgel. Figure 1f illustrates the inherent relationship between the viscosity of the TP-3DPgel precursor ink and shear rate. As the shear rate increases, the viscosity of the precursor inks transitions from high viscosity to low viscosity, exhibiting clear shear-thinning behavior. Notably, over the pH range from 3 to 7, the viscosity of TP-3DPgel precursor inks exhibited a trend of initial increase followed by a decrease. The obtained ink viscosity spans over 6 orders of magnitude (10^–2^ to 10^4^ Pa s^–1^). We conducted a comparative analysis of the TP-3DPgel designed using this innovative strategy with other advanced works that have been previously reported (Figure 1g).^30,35,38−45^ This comprehensive range of viscosities ensured the suitability of the TP-3DPgel precursor ink for diverse 3D printing applications, fully meeting the ink requirements for both DIW-3D and DLP-3D printing techniques. This adeptly addresses the “language” barriers arising from ink variations between different printing technologies, endowing TP-3DPgel with the potential capability to fabricate a variety of flexible electronic devices such as energy storage devices, flexible sensors, and electronic skin (e-skin).

**Figure 2:**
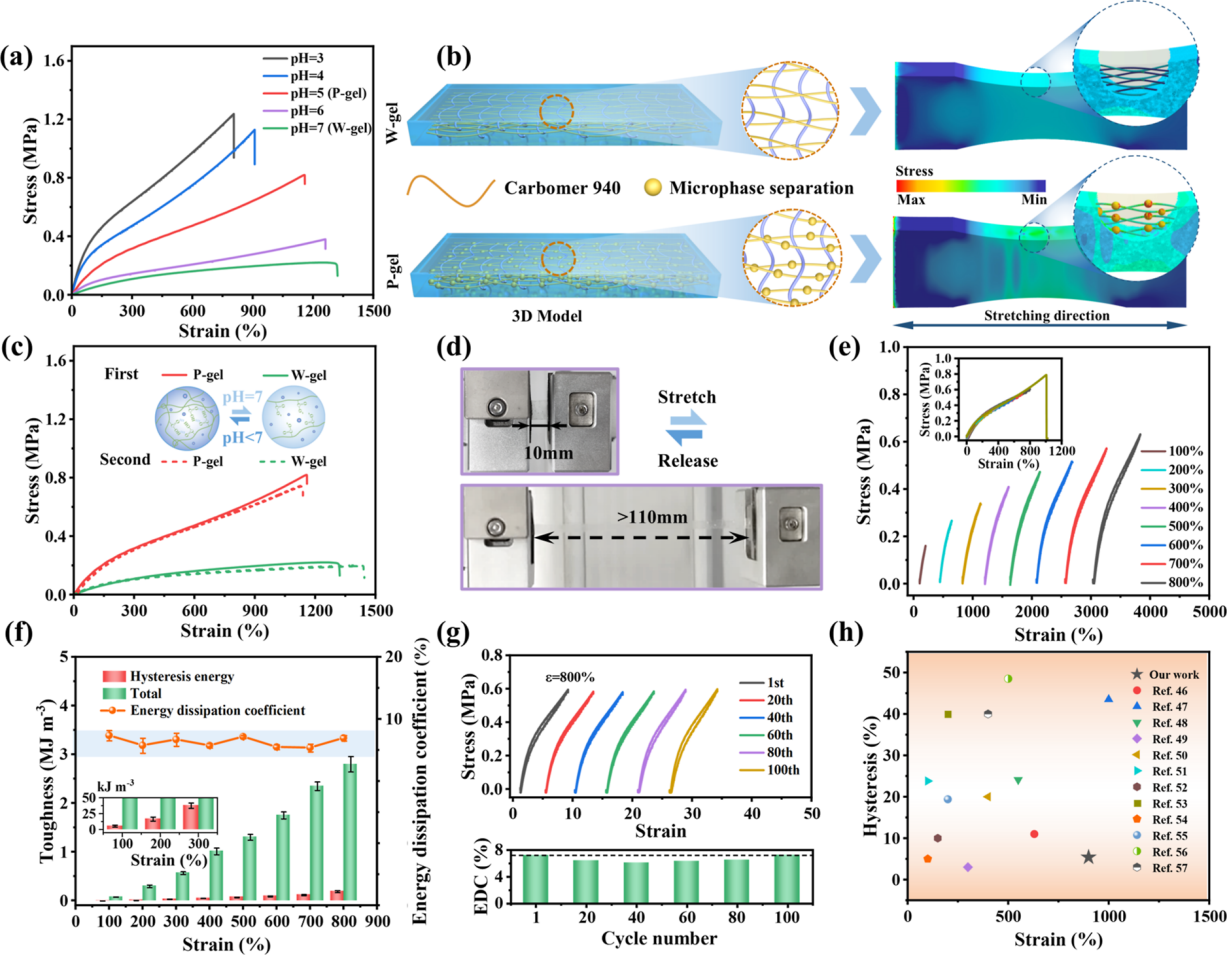
(a) Stress–strain curves of TP-3DPgel samples at different pH values. (b) FEA (stress distribution) of W-gel (pH = 7) and P-gel (pH = 5). (c) Impact of repeated establishment and disappearance of microphase separation on the mechanical properties of TP-3DPgel. (d) Optical images of TP-3DPgel under the tensile state. (e) Loading–unloading curves of TP-3DPgel prepared by DLP-3D printing at different strains (100%–800%), (f) along with the corresponding energy dissipation coefficients (EDCs) and toughness. (g) Mechanical curve of TP-3DPgel under continuous loading–unloading for 100 cycles at 800% strain, along with the corresponding EDC. (h) Rough comparison of strain and hysteresis the between this work and recently reported typical hydrogel.

We performed a series of uniaxial tensile experiments to quantify the ultimate strain and tensile strength of different microphase-separated TP-3DPgel. Initially, subsequent research subjects were determined by adjusting the ratio between the monomer (AAm) and the metal salt (CaCl_2_) solution, and the ratio of monomer to metal salt was finally determined to be 8:2 as the optimal component (for a detailed discussion of the process, please refer to the Supporting Information in Figure S9). Subsequently, Figure 2a demonstrated the mechanical properties of TP-3DPgel under different pH environments. It can be obtained from the stress–strain curves that TP-3DPgel exhibited stress weakening and strain enhancement with the increment of pH. In particular, the tensile strength and tensile strain of P-gel converted to W-gel transformed from 0.82 MPa and 1150% to 0.22 MPa and 1320%, respectively. The significant differences in the mechanical properties of TP-3DPgel further reveal the role of the microphase-separation region. Specifically, the microphase-separation structure within the P-gel functions as robust cross-linked regions during tensile deformation, effectively preventing the disentanglement of the network and the breakage of chemical bonds. This significantly enhanced the tensile strength of the hydrogel. These findings were consistent with the network configuration changes observed in the SEM analysis of TP-3DPgel. Figure 2b illustrates the process of establishing the finite element analysis (FEA) model. The results indicated that P-gel exhibited additional stress concentration compared to W-gel due to the presence of microphase separation regions under the same tensile strain simulation, which effectively validated the aforementioned observations. Interestingly, the mechanical properties of TP-3DPgel could be effectively tuned by adjusting the pH of the precursor solution (Figure 2c). We selected P-gel as the subject for further research due to its outstanding comprehensive properties, as it can undergo repeated loading–unloading cycles over a strain range exceeding 1000% (Figure 2d). Subsequently, we conducted cyclic loading–unloading experiments on specimens prepared by using P-gel. Excitingly, P-gel not only exhibited outstanding tensile strength but also demonstrated nearly ideal elastic behavior within a wide range of deformations. As illustrated in Figure 2e, P-gel undergone loading–unloading cyclic tests at various strains. Under deformation ranging from 100% to 800% strain, P-gel exhibited a purely elastic mechanical response, as evidenced by the loading–unloading curves displaying no hysteresis phenomenon. Even at an exceptionally high strain of 800% strain, the loading–unloading cyclic curve showed only minimal hysteresis loops. It was noteworthy that the loading–unloading cyclic curves across the 100% to 800% strain range perfectly coincide with stress–strain curves, further indicating the outstanding elasticity of the P-gel. We employed the energy dissipation method to assess the elasticity of P-gel and quantified its reversibility by evaluating the ratio (*T*) of the integrated area under the stress–strain curves during unloading and loading phases (*T* = ∫_0_^λ^σ(λ)_unloading_d(λ)/∫_0_^λ^σ(λ)_loading_d(λ)). The computational results are illustrated in Figure 2f, showing an increase in the toughness of P-gel with the rising strain, accompanied by a corresponding augmentation in hysteresis energy. Fortunately, the energy dissipation coefficient (EDC, 1-T) remained stable within the range of 5.4% to 7.4% under varying strains. Additionally, P-gel demonstrated a rapid self-enhancement performance over 100 consecutive loading–unloading cycles at 200% strain (Figure S10a). The corresponding stress–strain curve revealed negligible hysteresis loops and residual strain, while the calculated energy dissipation coefficient (Figure S10b) remained relatively stable. This phenomenon was similarly observed in the cyclic stretching performance at 400% and 800% strain (Figures 2g and S10c,d), showing the excellent fatigue resistance and cyclic stability of the P-gel. Throughout the entire experimental process, the specimen surfaces were coated with a moisturizing agent to mitigate the impact of dehydration on their mechanical performance. The tensile strain and hysteresis exhibited by the TP-3DPgel we prepared exceed those of the representative hydrogel reported in recent years (Figure 2h and Table S2).^46−57^

The synthesized TP-3DPgel exhibited outstanding 3D printing properties. In its initial P-gel state, it possessed excellent fluidity, making it well-suited for DLP-3D printing. Interestingly, although the neutralization process reduces the mechanical properties of TP-3DPgel (W-gel) by causing the dissociation of microphase separation domains, the resulting high viscosity rheological characteristics make it ideal for DIW-3D printing. Therefore, we believe that TP-3DPgel has the potential to become a universal 3D printing carrier to meet the needs of wearable devices, soft robotics, and biological tissues, among other fields.

**Figure 3:**
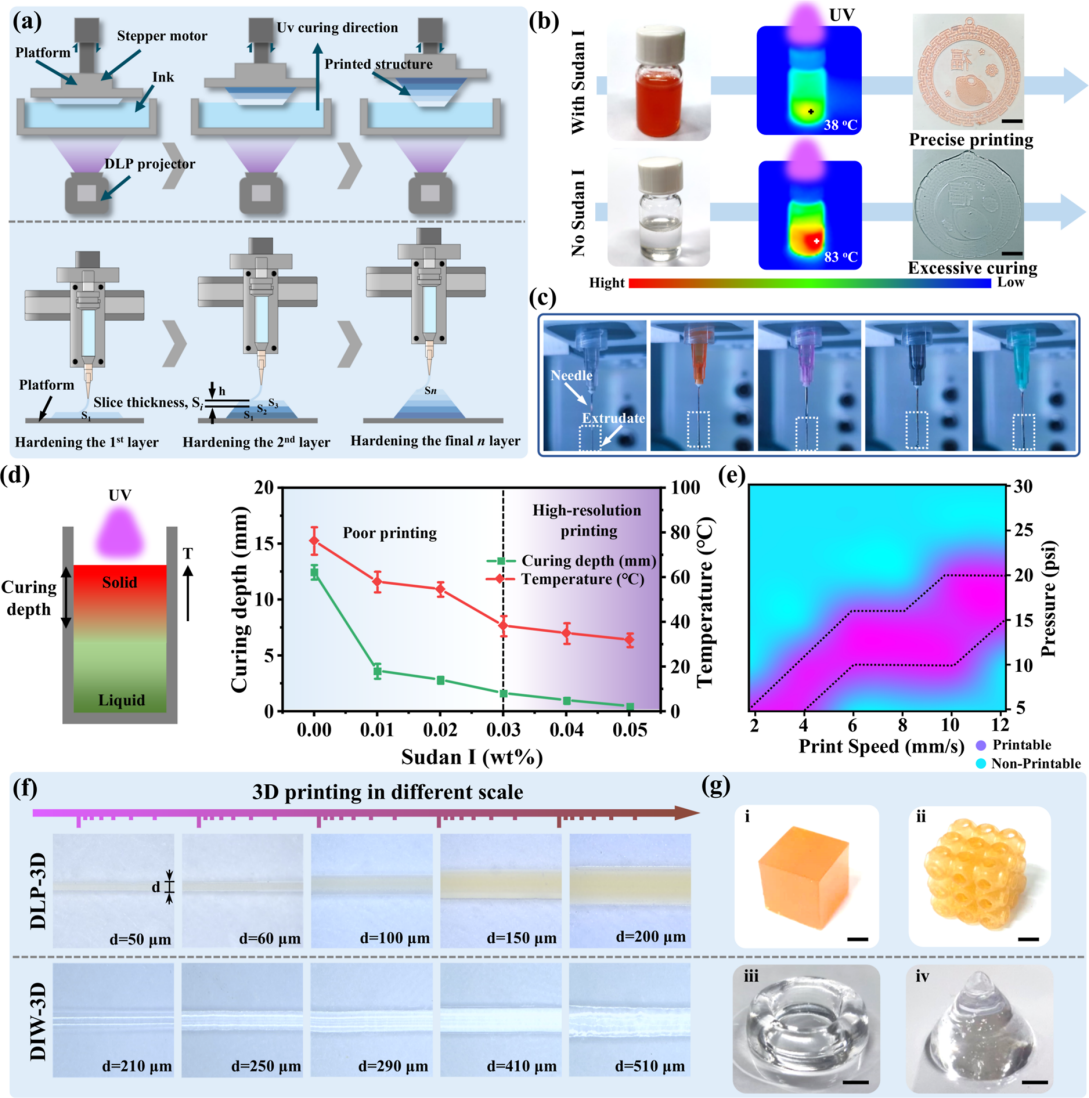
Molding quality of TP-3DPgel under different 3D printing strategies. (a) Process and mechanism of preparing TP-3DPgel devices via DLP-3D printing and DIW-3D printing. (b) Heat release rate of TP-3DPgel during photocuring is slowed down to enhance the resolution of DLP-3D printing by incorporating the light absorber Sudan I. Scale: 1 cm. (c) Optical images of fibers extruded by DIW-3D printing using nozzles of different sizes. (d) Diagram of the setup used to study the effect of Sudan I on the curing behavior of TP-3DPgel under ultraviolet light irradiation, and curing depth and polymerization-induced local temperature of TP-3DPgel as a function of Sudan I under UV illumination (power: 50 mW cm^–2^, and time: 30 s). (e) Printability corresponding to the printing pressure and printing speed of DIW-3D printing equipped with a 250 μm nozzle. (f) Producing TP-3DPgel fibers of multiscales through DLP-3D printing and DIW-3D printing. (g) 3D objects fabricated through DLP-3D printing and DIW-3D printing. Scale: 1 cm.

For printed devices, manufacturing precision was a key parameter for assessing the suitability and processability. First, DLP-3D printing was a typical top-down fabrication method. A stepper motor lowered the platform into the resin, and the entire 3D structure was fabricated by planning the path of 2D images at the bottom of the resin vat. Therefore, the ink used for DLP-3D printing needs good flowability. On the other hand, DIW-3D printing employed a bottom-up forming strategy. Ink was extruded from the syringe needle into filaments and shaped along predetermined routes. This presents certain challenges for the ink, as the extruded gel filaments need a sufficient self-supporting capability. Due to the different printing strategies of the two methods, the mechanisms affecting their printing precision cannot be generalized (Figure 3a).

The TP-3DPgel that we designed was a highly transparent ink with adjustable rheological properties. However, its high transparency became the potential factor affecting printing accuracy during DLP-3D. The addition of a light absorber to the TP-3DPgel could effectively mitigate the issue of excessive temperature caused by free radical polymerization, thereby preventing the adverse effects of excessive curing on printing accuracy. As depicted in Figure 3b, transparent TP-3DPgel underwent a polymerization reaction without the involvement of a light absorber, resulting in printed patterns exhibiting reduced precision due to overcuring, which is evidenced by the Chinese knot pattern printed. Similarly, the introduction of 0.03 wt % of Sudan I light absorber into TP-3DPgel resulted in printed patterns exhibiting clear structural contours within the preset sections, indicating the high-resolution DLP-3D printing capability of TP-3DPgel. To elucidate the potential impact of the light absorber on the DLP-3D printing accuracy of TP-3DPgel, we evaluated the curing depth and the temperature induced by polymerization under the same conditions (Figure 3d). The curing depth of TP-3DPgel significantly decreased as the amount of Sudan I increased, accompanied by a decrease in the temperature induced by polymerization from 83 ± 6.3 to 38 ± 3.1 °C. Therefore, the high-resolution DLP-3D printing capability of TP-3DPgel stemmed from the synergy between the selection of curing areas and heat dissipation, both of which were regulated by the content of the light absorber. We balanced time efficiency and manufacturing precision and chose 0.03 wt % of Sudan I as the light absorber to improve the 3D printing process. However, DIW-3D printing evaluates resolution by controlling the diameter of the nozzle to obtain TP-3DPgel filaments of different thicknesses. As shown in Figure 3c, we selected nozzles with diameters ranging from *d* = 210 μm to *d* = 510 μm (corresponding nozzle sizes are shown in Table S1) to print TP-3DPgel. The results demonstrate that the extruded gel filaments exhibit continuous linear behavior and have good shape retention capability. In subsequent DIW-3D printing processes, we balanced printing accuracy and manufacturing time by selecting a nozzle diameter of 250 μm for further experiments. Additionally, during the preparation of specimens using DIW-3D printing, TP-3DPgel is primarily influenced by printing speed and pressure. We defined a continuous filament print as a printable state, whereas other states, such as intermittent and excessive extrusion, were defined as nonprintable (Figure S11). Ultimately, the optimal range for DIW-3D printing was determined and shown in Figure 3e. Therefore, TP-3DPgel conformed to both additive manufacturing strategies and demonstrates potential for high-resolution shaping. Figure 3f shows optical images of TP-3DPgel filaments prepared using both printing methods at different scales, with filament diameters ranging from *d* = 50 μm to *d* = 510 μm, enabling the additive manufacturing of 3D structures such as cubes, triply periodic minimal surfaces: Schwarz P cell, rings, and cones. To our knowledge, this is an instance of multiscale 3D printing inks satisfying two different mechanisms that has been reported.

**Figure 4:**
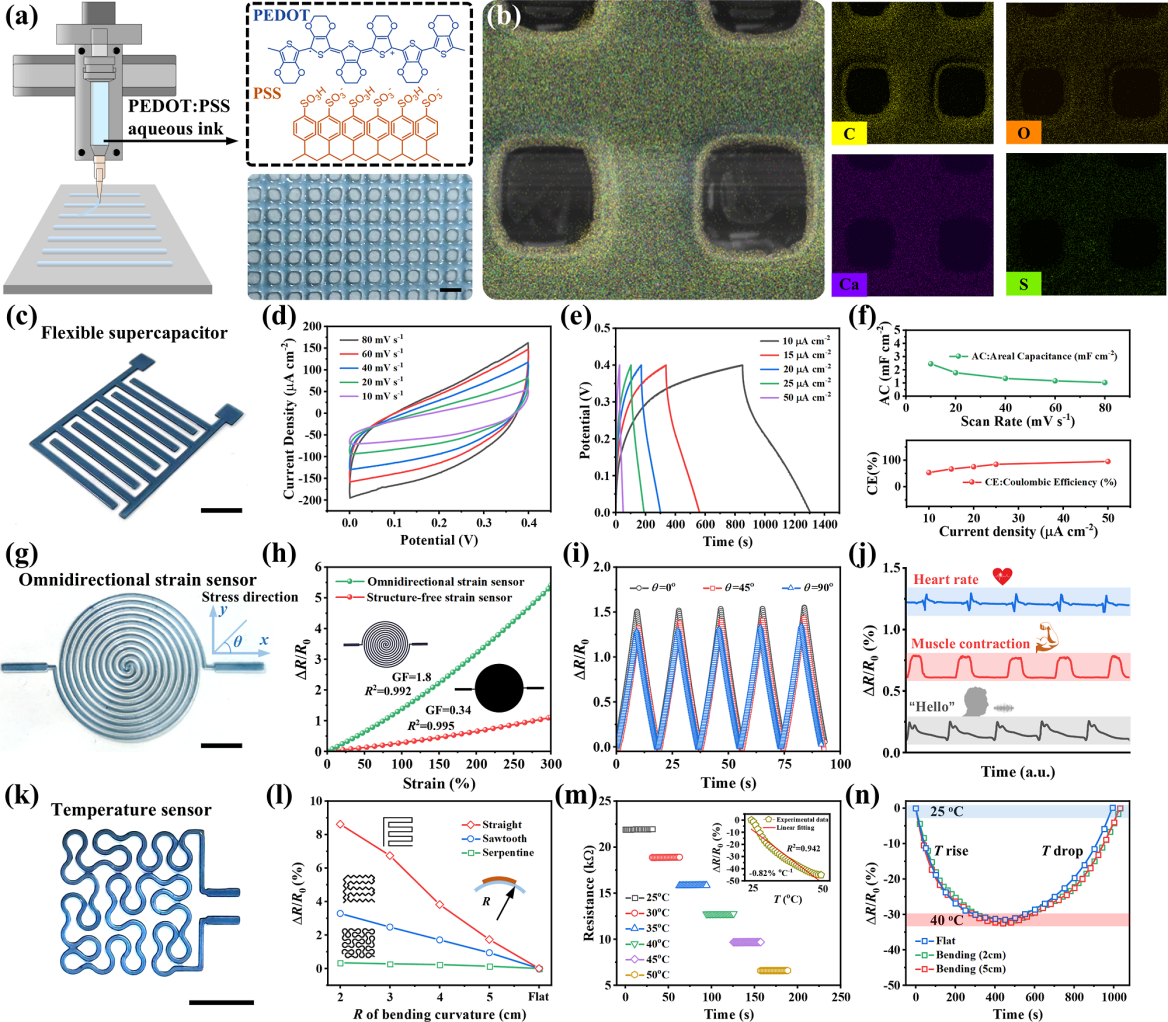
DIW-3D printing of TP-3DPgel flexible devices. (a) Schematic diagram of DIW-3D printing using PEDOT:PSS ink, along with the molecular formula of PEDOT:PSS, and microscopic images displaying the array of filaments. (b) Mapping images (C, O, Ca, and S) of TP-3DPgel. (c) Optical image of DIW-3D-printed flexible supercapacitors. (d) CV curves across various scan rates, (e) GCD curves across the current density range from 10 to 50 μA cm^–2^ (f) and along with the corresponding areal capacitance and Coulombic efficiency. (g) Optical image of DIW-3D-printed OS sensors, (h) followed by measuring their sensitivity, (i) signal response in different directions, and (j) application to physiological signals on various parts of the human body. (k) Optical image of DIW-3D-printed temperature sensors. (l) Resistance variation of temperature sensors based on different designs at different bending curvatures. (m) Dynamic response of the temperature sensor at different temperatures (*T*). (n) Response of the temperature sensor under mechanical deformation. Scale: 1 mm.

To demonstrate the widespread applicability of TP-3DPgel in DIW-3D printing, we conducted a series of manufacturing processes for flexible functional devices. The fabrication of flexible devices often involved the incorporation of various types of materials to impart the desired functional characteristics, such as PEDOT:PSS, MXene, carbon nanotubes (CNTs), and aramid nanofibers (ANFs). Among them, PEDOT:PSS has become one of the hot materials under research due to its excellent conductivity and flexibility. Therefore, we selected it to be incorporated into the ink to demonstrate the inclusiveness and processability of TP-3DPgel. As shown in Figure 4a, the incorporation of 0.5 wt % PEDOT:PSS into TP-3DPgel maintained excellent rheological properties and printing accuracy for DIW-3D printing ink (Figure S12). The lattice structure formed using DIW-3D printing technology allowed for clear identification of each filament outline, demonstrating the printing capability of TP-3DPgel. Furthermore, the uniform distribution of elements such as C, O, Ca, and S shown in the elemental mapping (Figure 4b) indicated the good dispersion of PEDOT:PSS in the ink, which provided opportunities for the subsequent fabrication of flexible devices.

First, we utilized TP-3DPgel mixed with PEDOT:PSS to print interdigitated electrodes, which were then assembled together with electrolyte to form a flexible supercapacitor (Figure 4c). As evident in Figure 4d, the detailed cyclic voltammetry (CV) curves exhibited quasi-rectangular characteristics and mirror-image symmetry at different scanning rates (10–80 mV s^–1^), indicating rapid charge transfer between the electrode and electrolyte. Figure 4e describes the GCD curves obtained at different current densities ranging from 10 to 50 μA cm^–2^, demonstrating quasi-triangular shapes for all curves, which further indicated excellent capacitance performance. Additionally, the areal capacitance corresponding to each curve decreased with increasing scan rate and reached 2.45 mF cm^–2^ at 10 mV s^–1^. Similarly, the Coulombic efficiency improved to 95.2% with an increasing current density (Figure 4f). Furthermore, we successfully fabricated an OS sensor using DIW-3D printing technology with mixed inks (Figure 4g). This complex circular grid structure provided the OS sensor with additional sensitivity and omnidirectional detection capability. Compared to the structure-free strain sensor, the OS sensor exhibited a sensitivity of up to gauge factor (GF) = 1.8 within a 300% strain range, while the latter only achieved GF = 0.34 (Figure 4h). Moreover, the OS sensor had the ability to perceive signals from omnidirectional mechanical stimuli, which maintained highly consistent relative resistance changes (Δ*R*/*R*_0_) in different directions (Figure 4i). Subsequently, we perfectly detected physiological signals such as heart rate, muscle contraction, and speech by attaching OS sensors to various parts of the human body (Figure 4j), demonstrating the potential applications of DIW-3D-printed OS sensors. In addition to their applications in energy storage and strain sensing, mixed inks were also 3D-printed into electronic skin temperature sensor devices (Figure 4k). In the electronic skin, temperature sensors based on mixed inks adopt a serpentine design that is insensitive to strain to withstand the stress effects of daily wear and tear (Figure 4l). As manifested in Figure 4m, the temperature sensor exhibits a negative temperature coefficient and stable temperature sensing performance within the physiological range of 25–50 °C, with a sensitivity of −0.82%/°C. Meanwhile, the temperature sensor attached to interfaces with different curvature radii presented the same temperature response curve, indicating that the shape has a negligible effect on temperature within a certain range of strain (Figure 4n).

In summary, we demonstrated the exceptional processability and inclusiveness of TP-3DPgel by 3D printing various functional flexible devices using DIW-3D printing. Our designed TP-3DPgel was not limited to the doping of PEDOT:PSS alone but was also compatible with the fusion with MXene, CNT, and ANF (Figure S13), which provided a reliable innovative strategy for the fabrication of flexible devices.

**Figure 5:**
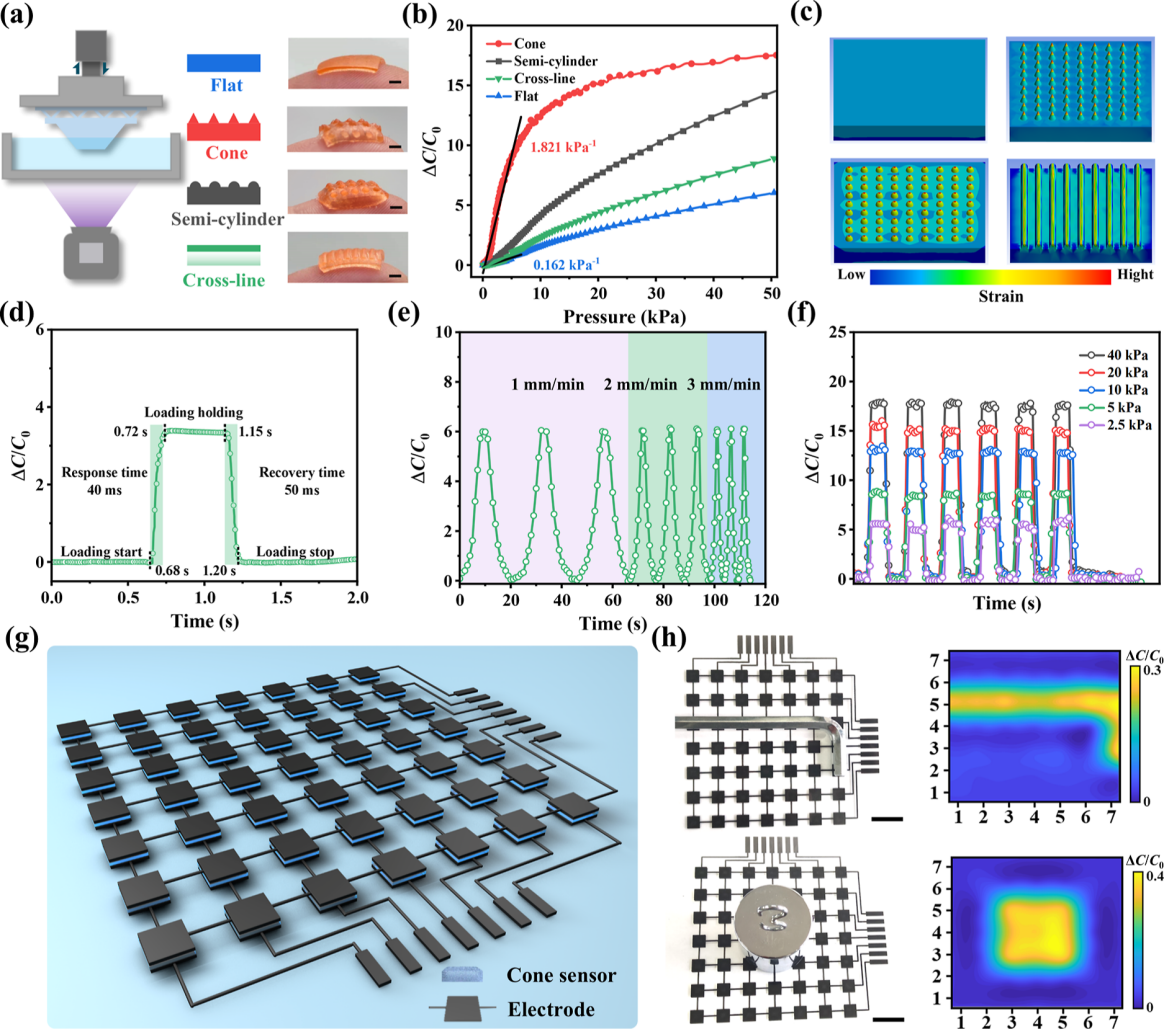
DLP-3D printing of TP-3DPgel piezocapacitive electronic skin. (a) DLP-3D printing and (b) sensitivity of TP-3DPgel sensors with different structures. Scale: 1 mm. (c) FEA strain cloud maps for TP-3DPgel sensors with different structures under the same load. (d) Response time, (e) velocity response, and (f) capacitance signal variations of cone-shaped TP-3DPgel sensors under different pressures. (g) Schematic diagram of integrated manufacturing of electronic skin array based on capacitive sensors using DLP-3D printing. (h) Pressure mapping cloud diagram obtained by placing different objects on the electronic skin. Scale: 2 cm.

We fabricated structured piezoelectric capacitive electronic skin (e-skin) with a microridge structure based on TP-3DPgel to demonstrate that its processability was also applicable to DLP-3D printing. As present in Figure 5a, different types of structured e-skin sensing units were perfectly replicated using DLP-3D printing. These intricately designed microstructures effectively enhanced the sensitivity of the piezoelectric capacitive e-skin. For example, while the sensitivity of the unstructured (flat) sensor was only 0.162 kPa^–1^ within the pressure range of 0–50 kPa, the sensitivity of the cone, semicylinder, and cross-line structures significantly increased under the same pressure conditions (Figure 5b). Among them, the cone pressure sensor exhibited the highest sensitivity of 1.821 kPa^–1^ due to the stress concentration effect. Figure 5c illustrates the deformation of different structural sensors under the same load (10 kPa) through FEA. The results demonstrated that the compressive deformation of structured sensors concentrates on the synapses, leading to the enhancement of sensitivity. We selected the cone sensor as the focus of the next study and obtained response and recovery times of 40 and 50 ms, respectively (Figure 5d), by applying pressure to reveal the sensor’s real-time information transmission capability. Furthermore, the rapid response mechanism of capacitive sensors avoided signal loss at high frequencies (Figure 5e), ensuring high-fidelity acquisition of capacitance changes at different speeds (1–3 mm min^–1^). As depicted in Figure 5f, the inherent structure and properties of the sensor allowed for the repeated recognition of capacitance signal changes under different pressures. It can accurately identify subtle pressures of 2.5 kPa and also provide consistent and reliable high-load (40 kPa) signal responses. Another attractive feature of TP-3DPgel for DLP-3D printing was its capability for the batch production of individual microdevices. We utilized this characteristic to in situ print sensor arrays on the expected circuitry to obtain e-skin with pressure recognition capabilities (Figure 5g). Subsequently, differently shaped metal objects were placed on the e-skin to detect capacitance changes caused by gravity. Correspondingly, the statistically processed capacitance signal cloud map can clearly identify spatial pressure changes generated by objects and accurately digitize the shape of the objects (Figure 5h). To further demonstrate the superiority of the TP-3DPgel in accommodating both DIW and DLP 3D printing, we used it to fabricate a flexible integrated device that meets the requirements for pressure sensing, strain sensing, and temperature sensing (Figure S14a). As shown in Figure S14b, the hybrid 3D-printed integrated device exhibits good flexibility and molding accuracy. Moreover, the flexible integrated device can detect subtle touches, wrist bending, and signal variations caused by temperature changes (Figure S14c–e), confirming that the TP-3DPgel is an ideal candidate for 3D-printed flexible devices.

## Conclusions

In conclusion, we reported a controlled phase separation strategy to regulate biphasic 3D-printed hydrogels, which were subsequently applied in the design and fabrication of flexible electronic devices. The strong intramolecular hydrogen bonding between polymer chains induces microphase-separated microstructures with clustered domains within the hydrogel. Notably, these microphase-separated regions exhibit reversible transitions through pH modulation, enabling the TP-3DPgel to achieve an extensive viscosity range from liquid to solid states. This adaptability overcomes the extreme rheological limitations associated with different 3D printing techniques. Moreover, the microphase-separated architecture imparted the TP-3DPgel with excellent mechanical properties, including high tensile strength and strain. Additionally, we demonstrated the hydrogel’s outstanding elasticity and fatigue resistance, evidenced by its ability to endure repeated loading and unloading cycles up to 100 times at 800% strain while maintaining minimal hysteresis. Furthermore, the optimized TP-3DPgel demonstrated outstanding molding accuracy suitable for both DIW and DLP-3D printing, successfully achieving a high-quality fabrication from 2D filaments to 3D models. Interestingly, we utilized the exceptional inclusiveness and processability of TP-3DPgel to fabricate various functional flexible devices, including energy storage, sensors, and e-skin, by using DIW and DLP-3D printing, respectively. From a broader perspective, our innovative design not only provides reliable strategies for the fabrication of functional devices through 3D printing but also creates interesting avenues for the functionalization and personalization of flexible devices.

## Experimental Section

### Materials

Acrylamide (AAm), carbomer 940, *N*,*N*′-methylene bis(acrylamide) (MBAA), calcium chloride (CaCl_2_), phenyl-2,4,6-trimethylbenzoylphosphinate (LAP), and photoinitiator 2959 were purchased from Tansoole; Sudan I was purchased from Shanghai Aladdin Biochemical Technology Co., Ltd.; and all chemicals were used as received without further purification. The others, including rubber, polyethylene terephthalate (PET), thermoplastic polyurethanes (TPUs), polytetrafluoroethylene (PTFE), copper (Cu) sheet, and aluminum (Al) plates, and glass plates were obtained from local suppliers.

### Fabrication of the TP-3DPgel and Ink

The 1 wt % carbomer solution was prepared by mixing an appropriate amount of carbomer 940 powder with DI water in a beaker at 500 rpm for a duration exceeding 12 h. The transparent liquid was prepared using a one-pot method by mixing 10 mL of carbomer solution, 0.1 wt % LAP or photoinitiator 2959, 1 wt % MBAA, and 30 g AAm. In the transparent solution, 20 wt %CaCl_2_ was incorporated as required. Among them, samples were directly cured under UV light (λ = 405 nm, with intensity of 50 mW cm^–2^) exposure to obtain raw materials for subtractive manufacturing. Additionally, 0.03 wt % of Sudan I is added to TP-3DPgel and stirred for 2 h to obtain TP-3DPgel ink for use in DLP-3D printing. By adding an appropriate amount of HCl or Ca (OH)_2_ solution to TP-3DPgel, TP-3DPgel inks with different pH values (3, 4, 5, 6, and 7) are obtained, among which the TP-3DPgel ink with pH = 7 is used for DIW-3D printing.

### 3D Printing of TP-3DPgel

After mixing the TP-3DPgel ink, it was subjected to ultrasonication for 10 min and then poured into the resin vat of a commercial DLP-3D printer. The three-dimensional model was imported into the accompanying software, and the slicing thickness was set to 30 μm with a projected light resolution of 50 μm and an exposure time of 30 s per layer. After printing completion, the specimens were separated from the curing platform and rinsed with anhydrous ethanol for 1 min to remove residual TP-3DPgel ink. Subsequently, the printed specimens underwent secondary curing in a UV curing (λ = 405 nm, with intensity of 50 mW cm^–2^) chamber for 1 h to enhance the mechanical properties of the printed parts. For DIW-3D printing, the corresponding TP-3DPgel ink was degassed in a planetary centrifugal mixer at a speed of 1000 rpm to remove bubbles and then transferred into the provided syringe. Different nozzle sizes are selected and connected to the syringe to achieve various printing resolutions. Import the three-dimensional model into the accompanying software and configure the relevant printing parameters, including a scanning speed of 2–12 mm/s, pressure ranging from 5 to 30 PSI, platform temperature set between 5 and 10 °C, and a slicing thickness of 0.2 mm. After printing is complete, the specimens are cured in a UV curing (λ = 405 nm, with intensity of 10 mW cm^–2^) chamber to obtain the final DIW-3D-printed devices. Unless specified otherwise, all specimens prepared via DIW-3D printing are printed using a nozzle with a size designation of 25G. We added 0.1 wt % PEDOT:PSS, 0.5 wt % MXene, 0.1 wt % CNT, and 0.5 wt % ANF into TP-3DPgel separately to obtain the corresponding DIW-3D printing doped inks, with printing parameters consistent with those mentioned above.

### Instruments and Measurement Methodology

Universal material testing machine (AG-X plus 50N, SHIMADZU) was used for mechanical testing of TP-3DPgel specimens. The prepared specimens were structurally characterized using Fourier transform infrared spectroscopy (FT-IR) with a wavelength range of 3000–3500 cm^–1^. The conductivity, CV curves, and galvanostatic charge–discharge (GCD) curves were executed on an electrochemical workstation (CHI660D). The tested specimens had dimensions of 10 mm × 10 mm with a thickness of 1 mm, clamped between two electrodes made of nickel mesh. The conductivity (σ) was calculated using the formula σ = *R*/(*L* × *A*), where *L* represents the thickness of the sample, *A* denotes the effective overlapping area between the sample and the electrodes, and *R* is the bulk resistance at the point where the imaginary part of impedance (*Z*″) equals zero on the Nyquist plot. The resistance and current signals of TP-3DPgel were measured by using a digital multimeter (Keithley, 2450). The sensitivity is defined as the GF, calculated as (Δ*R*/*R*_0_)/ε, where *R*_0_ is the initial resistance, Δ*R* is the change in resistance measured in real-time, and ε is the strain. Similarly, the sensitivity of pressure capacitive sensors and ion skins composed of TP-3DPgel is defined as δ = (Δ*C*/*C*_0_)/*P*, where *C*_0_ is the initial capacitance value and Δ*C* is the capacitance change corresponding to the pressure variation *P*. TP-3DPgel was tested for viscosity and shear rate using a rheometer; a steady-state flow test was performed with shear rates ranging from 0.01 to 1000 s^–1^. For FEA, the static analysis of the sensor was obtained by ANSYS simulation. The material parameters required for simulation were referenced from relevant parameters of rubber materials. The SAXS measurements of the TP-3DPgel samples were tested on the SAXS beamline (Xeuss 3.0, France) by using the Cu Kα radiation (λ = 1.54 Å), and the detector-to-sample distance is 1600 mm. The zeta potential of P-gel and W-gel suspensions was measured using a Zetasizer (BI-200SM).

## Supporting Information Available

Nyquist plot, optical and SEM images, FT-IR spectra, zeta potential distribution, SAXS patterns, mechanical curves of TP-3DPgel, different models of DIW-3D printing nozzles, rheological properties of P-gel before and after doping with PEDOT:PSS, model and dimensions of the DIW-3D printing nozzle, and rough comparison of strain and hysteresis between this work and the recently reported typical hydrogel (PDF)
