# Supplementary material for: Versatile Hydrogel Based on a Controlled Microphase-Separation Strategy for Both Liquid- and Solid-Phase 3D Printing
Source: ACS Nano. 2024 Oct 30;18(45):31148–59. doi: 10.1021/acsnano.4c08896 (PMC11688662; doi:10.1021/acsnano.4c08896)
Supplement: Supplementary file 1 — nn4c08896_si_001.pdf [file nn4c08896_si_001.pdf]

Supporting Information

A versatile hydrogel based on a controlled microphase-separation strategy for both liquid and solid phase 3D printing

Qirui Wu,[1,2] Yidan Xu,[3] Songjiu Han,[1,2] Anbang Chen,[1] Jiayu Zhang,[1] Yujia Chen,[1] Xiaoxiang Yang,[2] Lunhui Guan[*,1]*

[1.] State Key Laboratory of Structural Chemistry, Fujian Key Laboratory of Nanomaterials, and CAS Key Laboratory of Design and Assembly of Functional Nanostructures, Fujian Institute of Research on the Structure of Matter, Chinese Academy of Sciences, Fuzhou, Fujian 350108, China

[2.] School of Mechanical Engineering and Automation, Fuzhou University, Fuzhou 350108, China

[3.] Department of Oncology, The First Affiliated Hospital of Anhui Medical University, Hefei 230000, China.

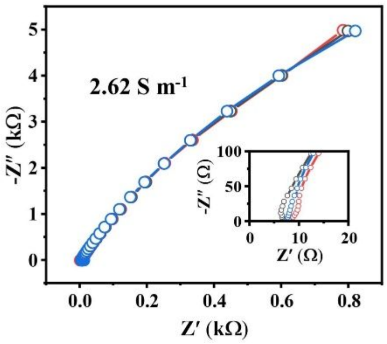

Figure S1. The Nyquist plot and conductivity of TP-3DPgel.

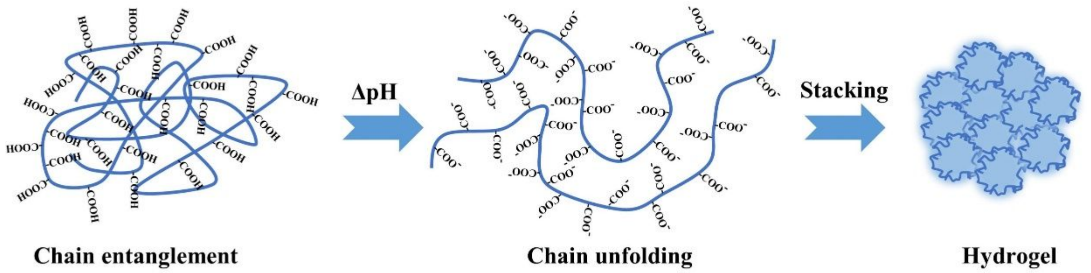

Figure S2. The TP-3DPgel forms a stable hydrogel network due to the repulsion of carboxylate anions.

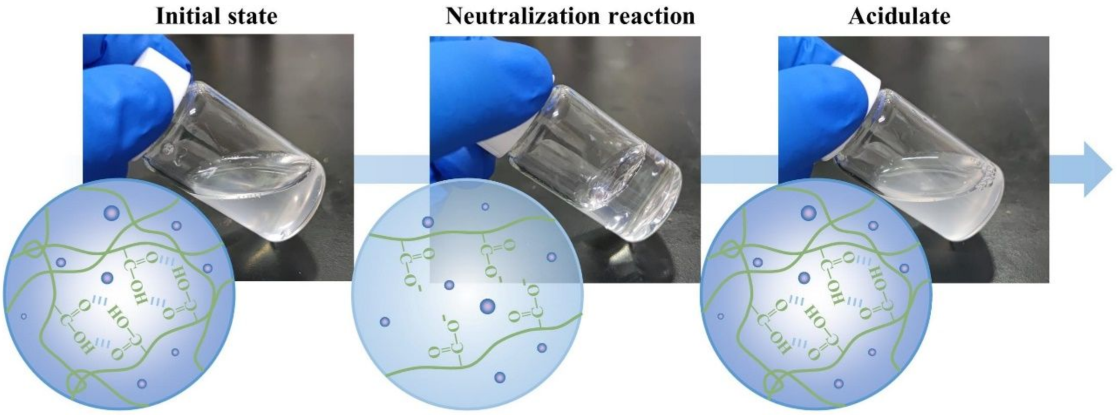

Figure S3. TP-3Dgel achieves reversible liquid-solid phase transition through pH adjustment.

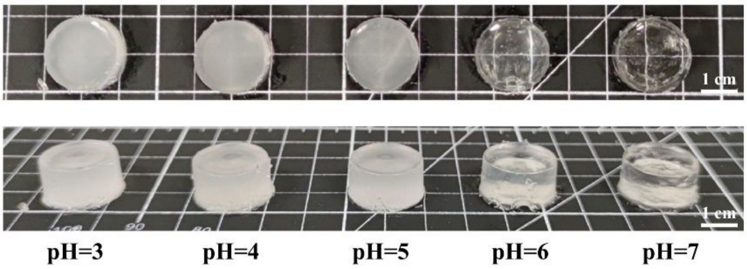

Figure S4. Optical images of TP-3DPgel at different pH values.

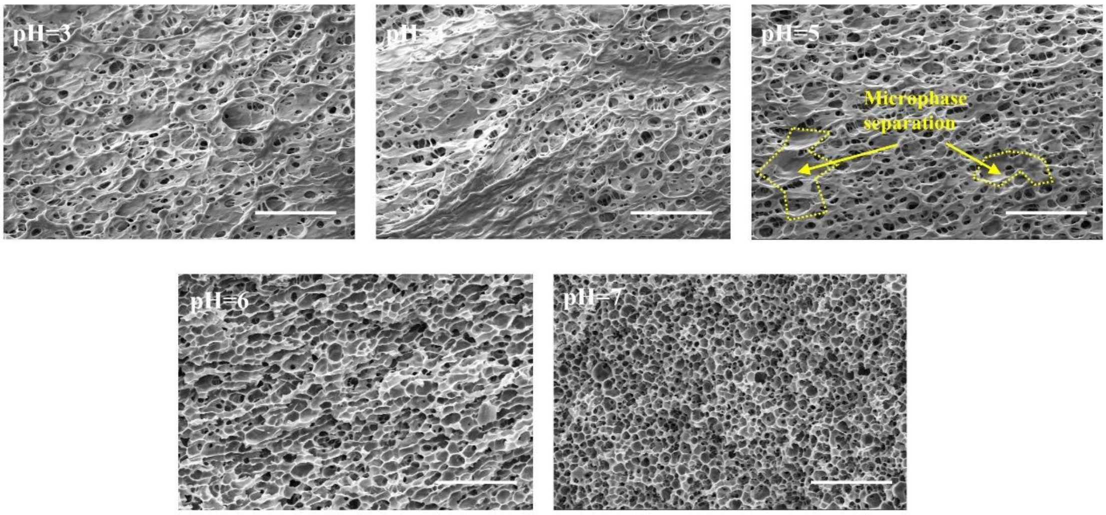

Figure S5. SEM images of TP-3DPgel at different pH values. The scale: 10 μm.

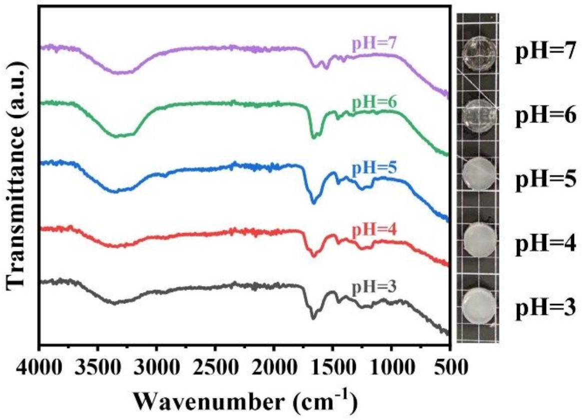

Figure S6. FT-IR spectra of TP-3DPgel at different pH values.

As featured in **Figure S6**, the characteristic peaks of TP-3DPgel at 1665 cm^{-1} and 1610 cm^{-1} were attributed to -C=O group stretching (Amide I, ν) and the N-H in-plane bending of $-CONH_2$ group (Amide II, δ), respectively. Simultaneously, the characteristic signal of TP-3DPgel at 1712 cm^{-1} correspond to the stretching vibration of -COOH groups. Notably, the characteristic peak at 1712 cm^{-1} disappeared, and the characteristic peaks at 1665 cm^{-1} and 1610 cm^{-1} severally shifted to 1650 cm^{-1} and 1550 cm^{-1} when the hydrogel transformed into W-gel, which further confirmed the dissociation of clustered -COOH groups on the polymer chains through hydrogen bonding interactions and the strong repulsion between anionic $-COO^-$ groups. [44, 45]

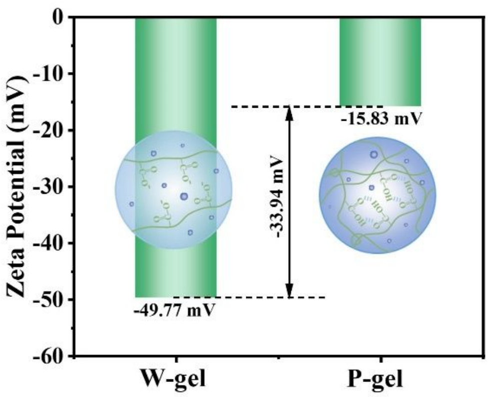

Measurement Parameters:

Mean Zeta Potential	= -49.77 mV	Liquid	= Water
Zeta Potential Model	= Smoluchowski	Temperature	= 22.0 癈
Mean Mobility	= -3.68 (µ/s) / (V/cm)	Viscosity	= 0.955 cP
pH	= 7.00	Refractive Index	= 1.331
Conductance	= 155 µS	Dielectric Constant	= 79.74
Concentration	= 2.00 mg/mL	Particle Size	= 315.0 nm

Measurement Parameters:

Mean Zeta Potential	= -15.83 mV	Liquid	= Water
Zeta Potential Model	= Smoluchowski	Temperature	= 22.0 癈
Mean Mobility	= -1.17 (µ/s) / (V/cm)	Viscosity	= 0.955 cP
pH	= 7.00	Refractive Index	= 1.331
Conductance	= 203 µS	Dielectric Constant	= 79.74
Concentration	= 2.00 mg/mL	Particle Size	= 315.0 nm

Figure S7. Zeta potential distribution of P-gel and W-gel.

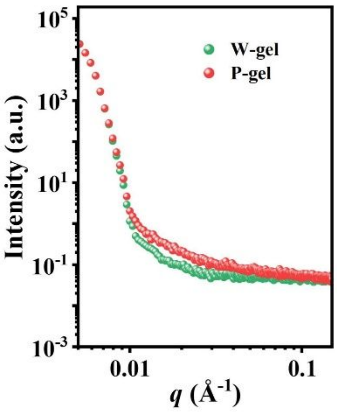

Figure S8. SAXS patterns of P-gel and W-gel, and corresponding intensity.

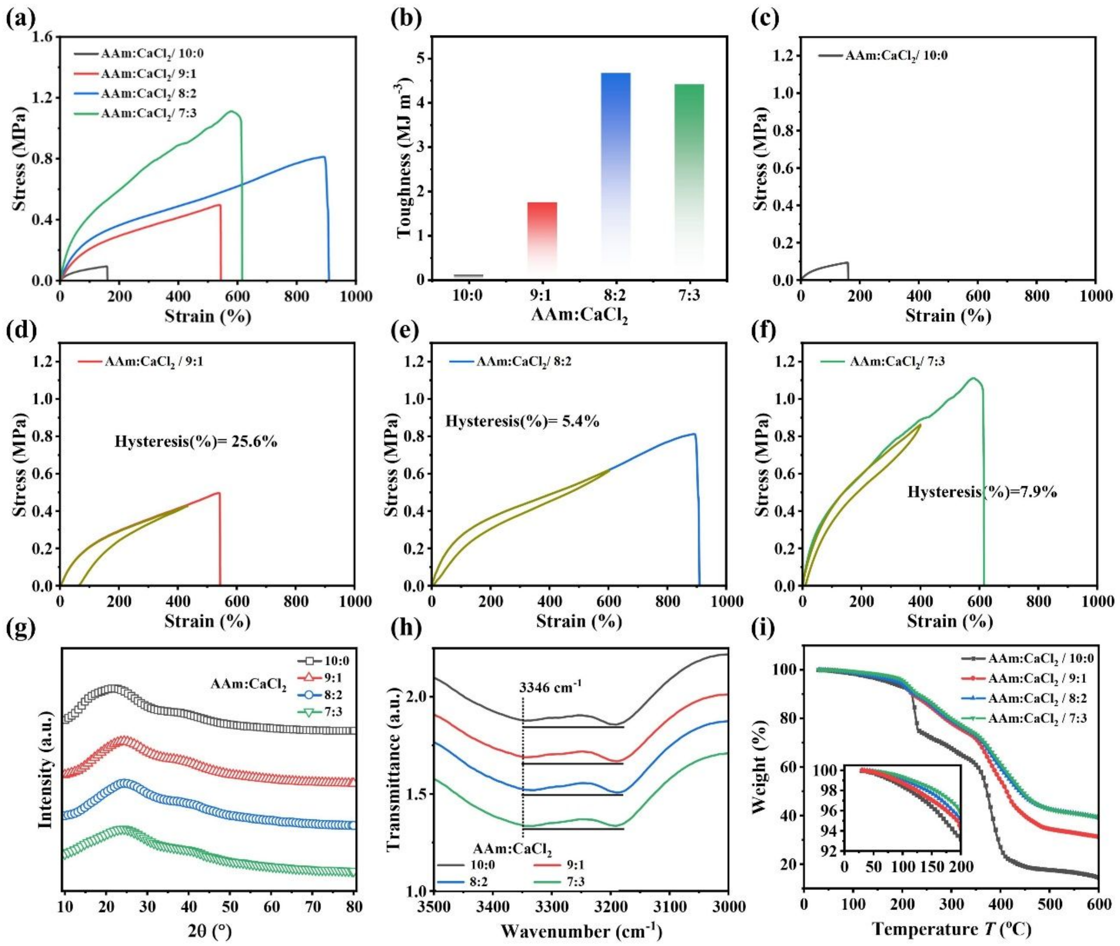

Figure S9. Mechanical curves of TP-3DPgel with different ratios of AAm and $CaCl_2$. (a) Stress-strain curves of TP-3DPgel at different CaCl2 concentrations, and (b) corresponding toughness at pH=5, (c-f) along with the corresponding loading-unloading curves and hysteresis. (g) X-ray diffraction (XRD) spectra of TP-3DPgel at different $CaCl_2$ concentrations. (h) FT-IR spectra of TP-3DPgel at different $CaCl_2$ concentrations. (i) Thermal gravimetric analysis of TP-3DPgel at different $CaCl_2$ concentrations.

Firstly, it is well-known that $CaCl_2$, as a metal salt, is introduced into the hydrogel polymer network to enhance mechanical properties. Therefore, it is essential to determine the ratio of the metal salt to the monomer when selecting the initial precursor solution for TP-3DP gel. We prepared specimens with $CaCl_2$ contents of 0, 10, 20, and 30 wt.%, and named them CPCa-0 (10:0), CPCa-1 (9:1), CPCa-2 (8:2), and CPCa-3 (7:3), respectively. As shown in **Figure S9(a)**, with increasing $CaCl_2$ content, both the tensile strain and fracture strength of TP-3DPgel were enhanced, exhibiting excellent mechanical performance in the CPCa-2 composition. Subsequent excessive introduction of $CaCl_2$ weakened the tensile properties of CPCa-3 while enhancing fracture strength. As shown in **Figure S9(b)**, the toughness values for

CPCa-0, CPCa-1, CPCa-2, and CPCa-3 are 0.11, 1.74, 4.67, and 4.41 MJ m^{-3}, respectively. More importantly, the hysteresis exhibited by CPCa-1, at 25.6%, is significantly higher compared to the 5.4% (CPCa-2) and 7.9% (CPCa-3) observed in other types of specimens **(Figure S9c-f)**. It was noteworthy that despite the deterioration in tensile properties caused by excessive $CaCl_2$, TP-3DPgel still maintains good resilience, further demonstrating that $CaCl_2$ was a key factor influencing mechanical property. This was mainly attributed to the physical interaction of Ca^{2+} with the polymer network of TP-3DPgel. Specifically, the molecular chains of AAm and PAA were interconnected with Ca^{2+} through metal coordination bonds and multiple weak hydrogen bonds in the TP-3DPgel system, thereby improving the crystalline domains formed by chain entanglements in the hydrogel, resulting in a denser polymer network and achieving excellent mechanical properties. **Figure S9(g)** presented the X-ray diffraction patterns of TP-3DPgel at different Ca^{2+} concentrations. The results indicated that the addition of Ca^{2+} causes a shift in the diffraction peak of the CPCa-0 from $2\theta=21.68^{o}$ to $2\theta=24.26^{o}$. Furthermore, the diffraction peak gradually becomes less pronounced as the Ca^{2+} content increases. This phenomenon confirms that Ca^{2+} interferes with the crystalline domains of the TP-3DP gel, thereby enhancing its mechanical properties. Similarly, microscopic transformations of TP-3DPgel were revealed through FT-IR spectroscopy. As shown in **Figure S9(h)**, in the TP-3DPgel system, hydroxyl group (-OH, *v*), carboxyl group (-COOH, *v*), and amide groups (-CO-NH_2, *v*) exhibited band broadening between 3000 and 3500 cm-1, attributed to stretching vibrations. Additionally, an increase in Ca^{2+} content leaded to stronger physical interactions between various components of the hydrogel, reflected in a significant enhancement of the 3346 cm^{-1} peak. Thermal gravimetric analysis (TGA) of different TP-3DPgel specimens revealed that as the Ca^{2+} content increases, a smaller weight loss was obtained within the temperature range of 200 ^{o}C (**Figure S9i**). This suggests that Ca^{2+}, through physical interactions with the polymer chains and water molecules, promoted the formation of a denser polymer network in TP-3DPgel, thereby restricting moisture evaporation in TP-3DPgel. In summary, we had reconstructed the polymer network structure at the microscopic level by adjusting the $CaCl_2$ content, endowing TP-3DPgel with excellent mechanical property characteristics. CPCa-2 (8:2) was selected as the primary sample for further investigation due to its outstanding comprehensive performance.

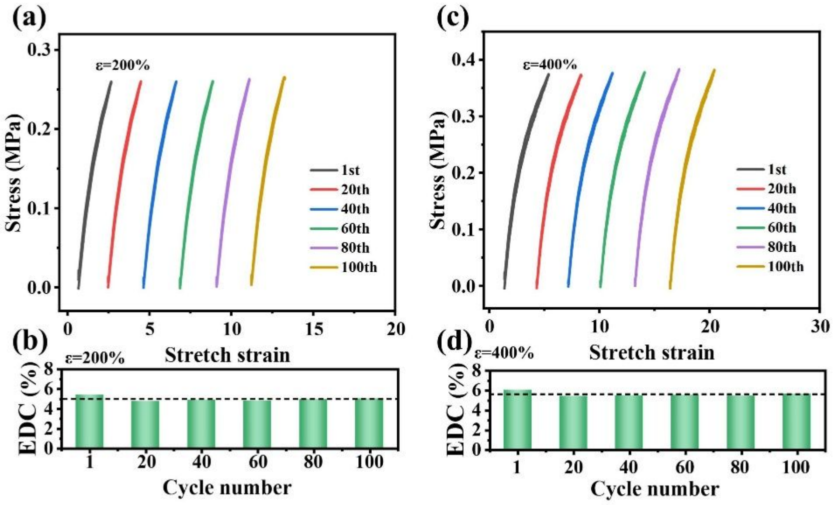

Figure S10. (a, c) Mechanical curves of TP-3DPgel prepared by DLP-3D printing under continuous loading-unloading cycles at 200% and 400% strains, (b, d) along with the corresponding energy dissipation coefficients.

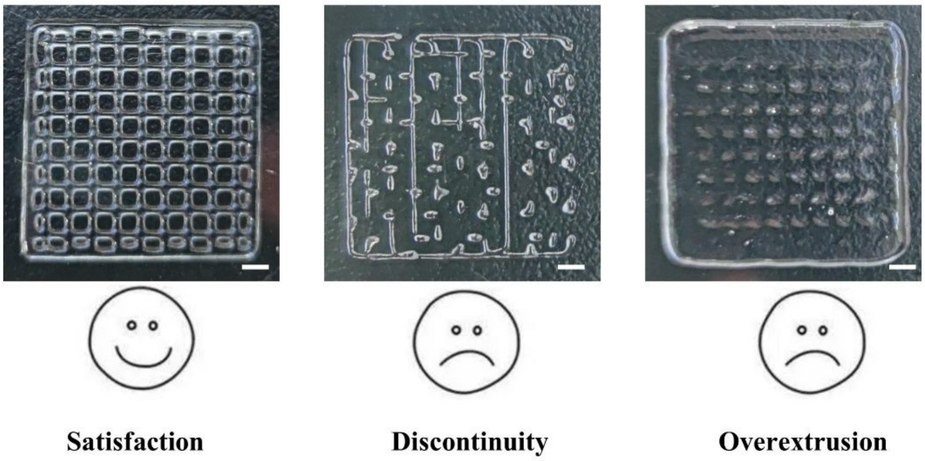

Figure S11. Criteria for evaluating the molding quality of DIW-3D printing.

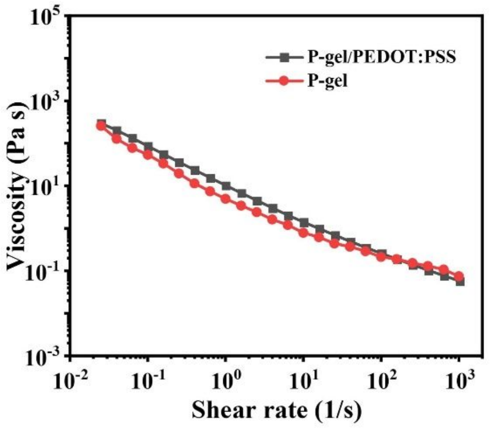

Figure S12. Rheological properties of P-gel before and after doping with PEDOT: PSS

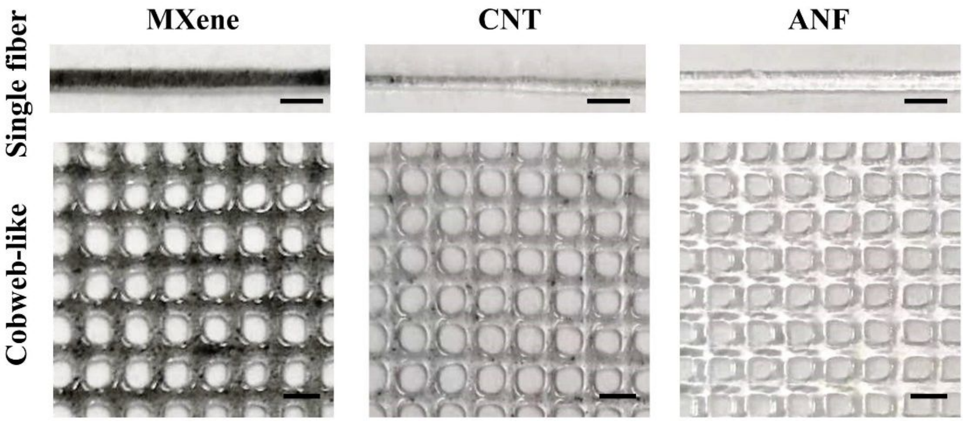

Figure S13. Optical images of TP-3DPgel encapsulating MXene, CNT, and ANF, and fiber arrays printed via DIW-3D printing. The scale: 1 mm.

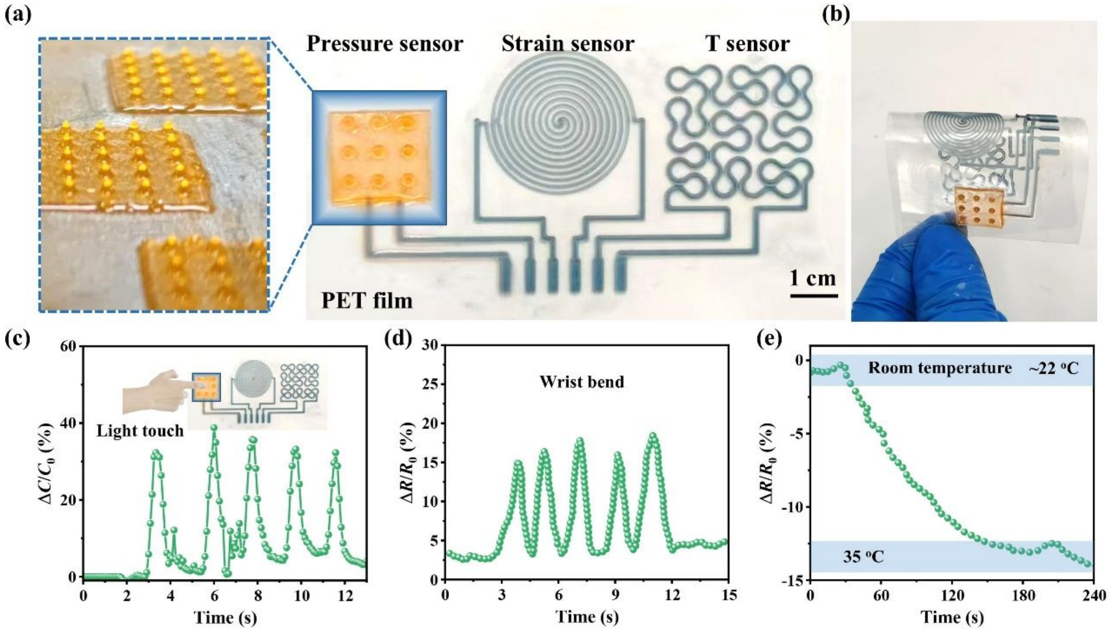

Figure S14. (a) Optical image of the flexible integrated device fabricated through DLP and DIW 3D printing, along with the corresponding (b) bending demonstration. Testing of various performances of the flexible integrated device: (c) pressure sensing; (d) strain sensing; (e) temperature sensing.

Table S1. The model and dimensions of the DIW-3D printing nozzle.

Size	Diameter/mm
27G	0.21
25G	0.25
23G	0.29
22G	0.41
21G	0.51

Table S2. A rough comparison of strain and hysteresis the between this work and recently reported typical hydrogel.

Materials	Strain (%)	Hysteresis (%)	References
PAA/AAm/$CaCl_2$	900	5.4	Our work
PEDOT: PSS/PAAMPSA/IL	630	11	[46]
PVA/PAMAA	1000	43.5	[47]
PVP/AA/ChCl	550	24.1	[48]
PEG/ChCl/AAm	300	3	[49]
HFBA/OEGA/[BMIM][TFSI]	400	20	[50]
CMFs/ChCl/AA	100	23.8	[51]
ChCl/VAM/AA/CMCS	150	10	[52]
MAAc/NPAM	200	40	[53]
IBA/MEA/LiTFSI	100	5	[54]
PVA/AMPS/AM/Gly	200	19.4	[55]
SPMA/MMA/Gly	500	48.5	[56]
OGE/AAm	400	40	[57]
